# Supplementary material for: Current practice patterns of osteoporosis treatment in cancer patients and effects of therapeutic interventions in a tertiary center
Source: PLoS One. 2021 Mar 11;16(3):e0248188. doi: 10.1371/journal.pone.0248188 (PMC7951835; doi:10.1371/journal.pone.0248188)
Supplement: Attachment — Submitted filename: Responce to Reviewer.docx [file pone.0248188.s003.docx]

November, 30, 2020

Dear Editor:

Thank you for checking the manuscript. We have summarized the corrections below based on your review. Thank you in advance for your kind help.

Best regards,

Nasa Fujihara

Additional Editor Comments:

1. Please check your MS against STROBE standards for cohort studies and add the STROBE checklist to your MS.

⇒ The checklist is attached at the end of the manuscript.

2. Please show p-values for table 1. My inspection of the table suggests that the treated/untreated groups differ. This should be checked explicitly, as it is an important potential source of confounding in your subsequent analysis.

⇒ We have included it because it affects the results of the analysis as you pointed out. Although there were fewer women in the untreated group, which may lead to a lower risk of fracture, the results actually showed a higher risk of fracture.

3. Date of entry into study should be explicitly defined, and survival curves plotted based on study entry, not diagnosis of osteoporosis. It is necessary to unpack those who were already being treated prior to cancer diagnosis.

⇒As you pointed out, the starting point was the time of participation in the study, and we have corrected this to include the notation in the graph.

Also, patients with a diagnosis of osteoporosis prior to the start of the study were excluded and this has been noted.

4. What about osteoporosis based on other low trauma fractures beside vertebral or femoral?

⇒ To clarify the definition, we only included the cases with vertebral or low-energy femoral fractures, or less than 70% of YAM value according to the Japanese diagnostic criteria of osteoporosis. So as you pointed out, the other low trauma fracture with high YAM value cases are not included. However, results includes all fragility fractures caused from low energy trauma.

Journal Requirements:

1. Please ensure that your manuscript meets PLOS ONE's style requirements, including those for file naming. The PLOS ONE style templates can be found at

⇒Done

2. Please provide additional details regarding participant consent. In the ethics statement in the Methods and online submission information, please ensure that you have specified (1) whether consent was informed and (2) what type you obtained (for instance, written or verbal, and if verbal, how it was documented and witnessed). If your study included minors, state whether you obtained consent from parents or guardians. If the need for consent was waived by the ethics committee, please include this information.

⇒ I have changed the manuscript following above.

3. We note that you have indicated that data from this study are available upon request. PLOS only allows data to be available upon request if there are legal or ethical restrictions on sharing data publicly. For information on unacceptable data access restrictions, please see http://journals.plos.org/plosone/s/data-availability#loc-unacceptable-data-access-restrictions.

In your revised cover letter, please address the following prompts:

b) If there are no restrictions, please upload the minimal anonymized data set necessary to replicate your study findings as either Supporting Information files or to a stable, public repository and provide us with the relevant URLs, DOIs, or accession numbers.

⇒Done
