# Supplementary material for: Current practice patterns of osteoporosis treatment in cancer patients and effects of therapeutic interventions in a tertiary center
Source: PLoS One. 2021 Mar 11;16(3):e0248188. doi: 10.1371/journal.pone.0248188 (PMC7951835; doi:10.1371/journal.pone.0248188)
Supplement: Attachment — Submitted filename: Responce_to_Reviewers_0203.docx [file pone.0248188.s004.docx]

*February 3, 2021*

Dear Dr. Blank,

Academic Editor

*PLOS ONE*

Dear Editor:

We would like to thank you for your response and for giving us the opportunity to improve and resubmit our manuscript (PONE-D-20-33343R1) entitled "**Current practice patterns of osteoporosis treatment in cancer patients and effects of therapeutic interventions in a tertiary center**." We are hereby resubmitting a revised manuscript conforming to all of the reviewers’ comments. In particular, we have addressed all the reviewers’ comments in a point-by-point manner and revisions are indicated in red font in the revised manuscript. We hope that the revised manuscript is now suitable for publication in your journal.

Thank you for your consideration. I look forward to hearing from you.

Sincerely,

Nasa Fujihara,

Section of Orthopedic Surgery, Aichi Cancer Center

1-1 Kanokoden Tikusa-ku,

Nagoya City, Aichi, Japan 464-8681

Email: nfujihara@aichi-cc.jp

Phone: 052-762-6111

FAX: 052-762-6111

**Reviewer #1:** The result is somehow expected where those being treated should have a lower incidence of fragility fracture. It is supported by this piece of research. 9% of the patients were being treated with Vitamin D. Many current studies showed the important role of Vitamin D in patients with cancer especially breast cancer. Is Vitamin D considered a treatment for osteoporosis in patient with cancer in your centre? What kind of Vitamin D was being used? Was it single therapy or in combination with anti-osteoporosis medicines? Did you look at the vitamin D level of your patients, both baseline and after treatment?

Although it was mentioned in your document that there might be a role of teriparatide in treating cancer patients with osteoporosis, it is clearly stated in the teriparatide product information that it should not be used in cancer patients with skeletal metastatic lesion. What is your view on this?

**Response**

We would like to thank the reviewer for evaluating our manuscript and for these constructive comments.

First, regarding vitamin D, we did use not the natural form of vitamin D that is often discussed nowadays. “Vitamin D” described in this study was actually “eldecalcitol” or “alfacalcidol,” which is an active vitamin D3 prodrug. Besides, we do not have a specific hospital or medical department protocol for applying Vitamin D drugs. Probably, the use of Vitamin D has been reported in some cases of breast cancer, as the reviewer stated.

However, in Japan, “eldecalcitol” or “alfacalcidol” is often prescribed as an initial drug for osteoporosis treatment. Please note that we have edited the text and figures to make it clear that we used an active vitamin D3 prodrug. Especially, we have added the following sentences in the revised manuscript:

“An active vitamin D3 form, the one most commonly prescribed in general practice, was used in 9% of all cases.” (Lines 137–138)

“The active form of vitamin D3 included eldecalcitol and alfacalcidol.” (Line 147)

Moreover, teriparatide's contraindication for cancer patients with skeletal metastatic lesion is mainly based on past studies showing that teriparatide increased osteosarcoma in rats. However, in this study on rats, the used doses were dozens of times higher and provided for a longer period of time than those given to humans. Therefore, there are many arguments regarding whether it can directly apply to humans. In fact, as a recent study that analyzed the data of patients from a US database did not show any evidence concerning the role of teriparatide in increasing osteosarcoma or malignant bone tumors, we believe that the indications for teriparatide will be expanded in the future. Alternatively, another new osteoporosis drug, romosozumab, is indicated for cancer patients and may be a useful treatment option. Please note that we have included these issues in the discussion of the revised manuscript as follows: “However, recent research has provided new evidence concerning the use of teriparatide (25, 30, 31). Interestingly, Gilsenan et al. reported that the incidence of osteosarcoma associated with teriparatide use during a 15-year period was not different than what was expected based on the background incidence rate of osteosarcoma after examining data from a US database (30). Besides, other new agents, such as romosozumab, humanized IgG2 monoclonal antibody, or annual zoledronic acid, were allowed to be used for the treatment of cancer patients. These relatively new drugs will be used more aggressively for such patients in the future.”

(Lines 256–263)

**Reviewer #2:** The paper is scientifically sound and presents useful information. However, the written language used in the manuscript is not appropriate for a scientific publication (For example: first person used not be in scientific manuscript.

**Response**

We would like to thank the reviewer for evaluating our manuscript and for this comment. Please note that we have sent our manuscript to an English editing company (Editage) for English proofreading. We hope that the level of English has significantly improved in the revised manuscript.
